# Method for the execution of skin tests in young pediatric patients with suspected allergy to milk and egg proteins

**DOI:** 10.1186/2045-7022-1-S1-P94

**Published:** 2011-08-12

**Authors:** Elisa Panfili, Arianna Latini, Giulia Maria Campus, Miriam Castagnino, Emanuele Belardi, Francesco Marcucci

**Affiliations:** 1 University of Perugia, Department of Medical Surgical Specialty and Public Health, Perugia, Italy

## 

Prick tests, which are used on children up to the age of 2 in order to diagnose a suspected food allergy to milk and egg proteins, are difficult to carry out due to their lack of compliance. In order to overcome this problem, we have put into practice a method allowing to accurately carry out skin tests and reducing thereby by a single act the immobilization time.

The method is based on the creation of a device which contains 3 needles 3 cm away from each other for testing 2 allergens, milk and egg white, and histamine at 1% from Stallergenes.

The device we have created to perform the skin test is made up of a base in which centre the 3 needles for the prick test are fixed in sequence. The base comes with a handle to be held by the health worker to perform the multi-test on the forearm perpendicularly.

In 18 children whose age ranges from 2 to 24 months already positive to egg and/or cow’s milk and in 10 negative skin tests children, the abovementioned allergens and the histamine were carried out with the traditional method and with the new one within a span of one week from each other. With the two methods 3 children were positive to milk and to egg, 5 only to milk and 10 only to egg in a perfectly identical way; 10 healthy controls were negative in all cases. By comparing the diameter of the wheals we observed that the results were completely superimposable.

Based on our findings, we can say that the method we developed has the same sensitivity and specificity as the traditional method, with the advantage of being easier to carry out because it works faster and less painfully (1-2 sec) for these patients.

